# Survival impact of perioperative changes in prognostic nutritional index levels after esophagectomy

**DOI:** 10.1007/s10388-021-00883-5

**Published:** 2021-09-21

**Authors:** Ryoma Haneda, Yoshihiro Hiramatsu, Sanshiro Kawata, Junko Honke, Wataru Soneda, Tomohiro Matsumoto, Yoshifumi Morita, Hirotoshi Kikuchi, Kinji Kamiya, Hiroya Takeuchi

**Affiliations:** 1 Department of Surgery, Hamamatsu University School of Medicine, Hamamatsu, Japan; 2 Department of Perioperative Functioning Care and Support, Hamamatsu University School of Medicine, 1-20-1 Handayama, Higashi-ku, Hamamatsu, Shizuoka 431-3192 Japan

**Keywords:** Esophageal cancer, Esophagectomy, Prognostic nutritional index

## Abstract

**Background:**

The correlation between perioperative changes in nutritional status during esophagectomy and prognosis remains unclear. This study aimed to evaluate the impact of changes in prognostic nutritional index levels during the perioperative period on esophageal cancer patient survivals.

**Methods:**

From January 2009 to May 2019, 158 patients with esophageal squamous cell carcinoma were enrolled. From the time-dependent ROC analysis, the cutoff values of preoperative and postoperative prognostic nutritional index levels were 46.9 and 40.9. Patients were divided into preoperative-high group (Group H) and preoperative-low group (Group L). Then, patients in Group L were divided into preoperative-low and postoperative-high group (Group L–H) and preoperative-low and postoperative-low group (Group L–L). Long-term outcomes and prognostic factors were evaluated.

**Results:**

Patients in Group L had significantly worse overall survival than those in Group H (*p* = 0.001). Patients in Group L–L had significantly worse overall survival than those in Group L–H (*p* = 0.023). However, there was no significant difference in overall survival between Groups H and L–H (*p* = 0.224). In multivariable analysis, advanced pathological stage (hazard ratio 10.947, 95% confidence interval 2.590–46.268, *p* = 0.001) and Group L–L (hazard ratio 2.171, 95% confidence interval 1.249–3.775, *p* = 0.006) were independent predictors of poor overall survival.

**Conclusions:**

Patients in Group L–H had a good prognosis, similar to those in Group H. This result indicated that increasing the postoperative prognostic nutritional index level sufficiently using various intensive perioperative support methods could improve prognosis after esophagectomy in patients with poor preoperative nutritional status.

**Supplementary Information:**

The online version contains supplementary material available at 10.1007/s10388-021-00883-5.

## Introduction

Esophageal cancer is the sixth leading cause of death from cancer worldwide [1]. Despite the development of multimodal therapies, its high malignant potential and poor prognosis persist as serious problems. Transthoracic esophagectomy has been recognized as a standard treatment for esophageal squamous cell carcinoma (ESCC) [2–4]. However, it is a highly invasive surgical procedure that results in a systemic inflammatory response and poses a risk of postoperative complications [5]. Postoperative complications may induce nutritional deficiencies and organ disorders [6, 7]. Studies have revealed that nutritional support is important to reduce the incidence of postoperative complications and shorten hospital stays [8, 9]. Thus, the baseline immune-nutritional status in cancer patients plays a significant role in survival.

Recently, nutritional or inflammatory status markers have been identified [10, 11]. The prognostic nutritional index (PNI), calculated based on the serum albumin level and total lymphocyte counts, reflects both inflammatory and nutritional status [12]. Previous reports have revealed that preoperative-low PNI level was associated with worse survival in patients with ESCC [13, 14]. However, the relationship between changes in the PNI levels from the pre- to postoperative phase and survival remains unclear.

In this study, we hypothesized that the recovery of PNI level after esophagectomy may improve patient prognosis. The correlation between nutritional and immunological status during esophagectomy and prognosis was investigated using PNI in patients with ESCC.

## Patients and methods

### Patients

From January 2009 to May 2019, 188 patients with ESCC at the Department of Surgery, Hamamatsu University School of Medicine, were retrospectively reviewed. All patients underwent esophagogastroduodenoscopy (EGD) and computed tomography (CT) from the neck to the pelvis to determine the clinical stage. The clinical stage was diagnosed based on the Union for International Cancer Control TNM classification of malignant tumors, 8th edition [15].

**Fig. 1:**
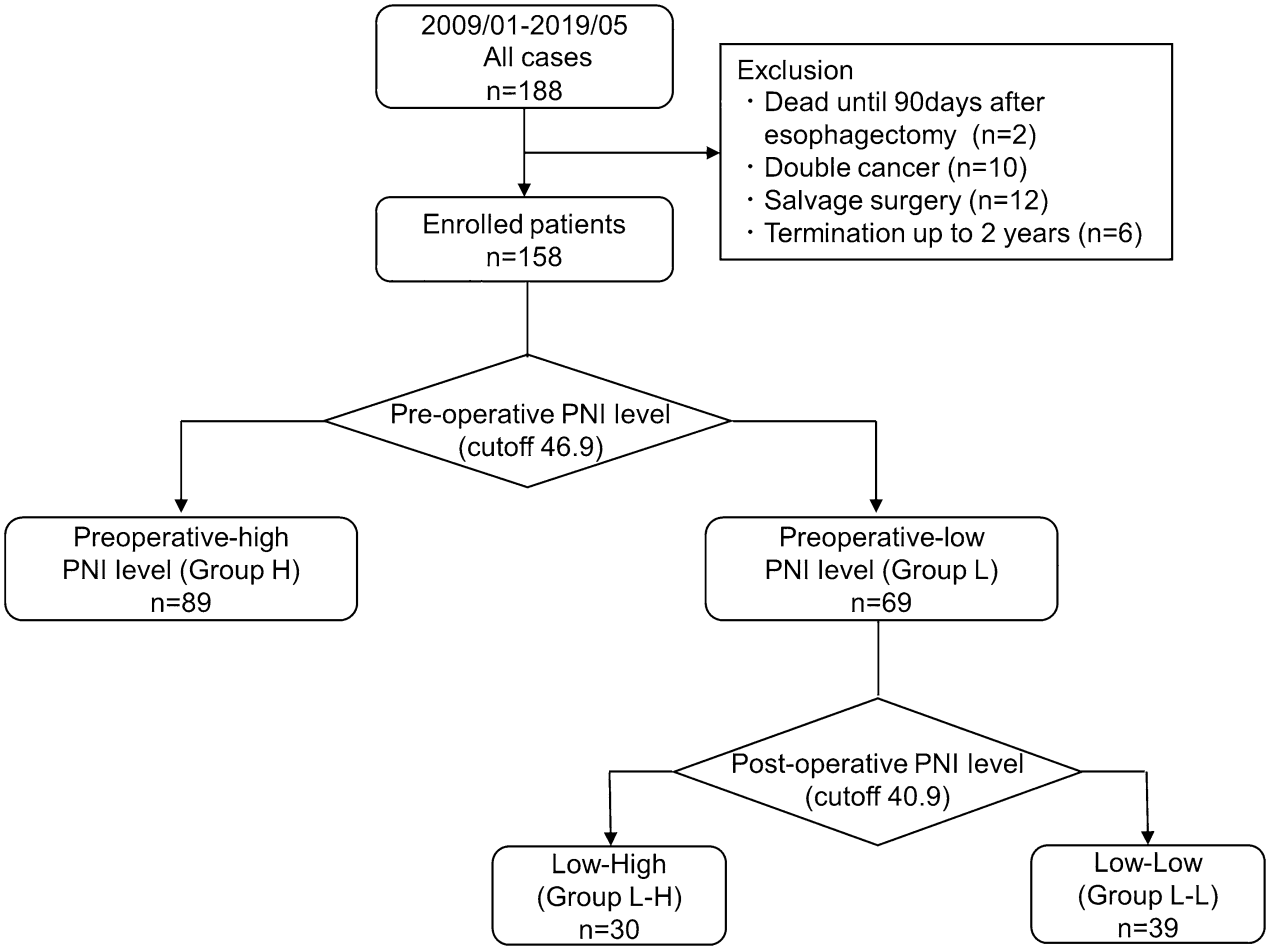
Flow diagram of the study. Patients enrolled in this study were classified into preoperative-high PNI group (Group H) and preoperative-low PNI group (Group L). Furthermore, based on the postoperative prognostic nutritional index levels, patients in Group L were divided into preoperative-low and postoperative-high group (Group L–H) and preoperative-low and postoperative-low group (Group L–L)

Patients who met the following criteria were enrolled in this study: (1) age > 20 years, (2) Eastern Cooperative Oncology Group performance status of 0–1, (3) histological diagnosis of ESCC by endoscopic biopsy, (4) no double cancer, (5) radical esophagectomy, (6) survival for at least 90 days after surgery, and (7) patients who survived and were followed up for more than 2 years. Patient ineligible for study enrollment was based on the following exclusion criteria: death within 90 days after surgery (*n* = 2), double cancer (*n* = 10), salvage surgery (*n* = 12), and interruption of follow-up within 2 years (*n* = 6). Finally, 158 patients were included in the study (Fig. 1).

### Determination of the cutoff values for the prognostic nutritional index

PNI was calculated as 10 × serum albumin (g/dL) + 0.005 × total lymphocyte counts (per mm^3^) [12]. Preoperative PNI levels were measured just before the surgery. When patients were administered neoadjuvant chemotherapy, the PNI level was measured after this treatment. Postoperative PNI levels were obtained 1 month after surgery. The time-dependent receiver operating characteristic (ROC) analysis of preoperative PNI-based prediction of 5-year survival revealed that the cutoff value of preoperative PNI was 46.9 (sensitivity = 0.613, specificity = 0.651, Youden index = 0.264) (Online Resource 1). Moreover, the time-dependent ROC analysis of postoperative PNI-based prediction of 5-year survival revealed that the cutoff value of postoperative PNI was 40.9 (sensitivity = 0.572, specificity = 0.540, Youden index = 0.112) (Online Resource 2). Based on these results, patients were divided into preoperative-high group (Group H) and preoperative-low group (Group L). Furthermore, patients in Group L were divided into preoperative-low and postoperative-high group (Group L–H) and preoperative-low and postoperative-low group (Group L–L) (Fig. 1).

### Treatment and postoperative complications

Neoadjuvant chemotherapy was performed as a standard treatment for patients with non-stage I ESCC. The treatment regimen was a combination of cisplatin and 5-fluorouracil or a combination of docetaxel, cisplatin, and 5-fluorouracil. Transthoracic esophagectomy with 2- or 3-field lymph node (LN) dissection and gastric conduit reconstruction via the posterior mediastinal route was performed as a standard surgical procedure at our institution [16]. In the thoracic approach, video-assisted thoracoscopic surgery in the left decubitus position was generally adopted. Thoracotomy was performed for patients who refused thoracoscopy or were enrolled in another clinical trial. When the stomach could not be used due to a previous history of gastrectomy, reconstruction using the right hemi-colon was performed [16]. Only one patient in Group L–H underwent reconstruction using the right hemi-colon in a two-stage operation because of a history of gastrectomy due to gastric ulcer and scleroderma. Postoperative complications were evaluated for pneumonia, anastomotic leakage (AL), and surgical site infection (SSI) using the Clavien–Dindo classification. Complications of grade 2 or higher were identified as postoperative complications [17, 18]. The multidisciplinary support team, comprised surgeons, physicians of rehabilitation, nurses, dentists, physiotherapists, speech–language–hearing therapists, and managerial dieticians. This team was set up since April 2017, to prevent complications and ameliorate the postoperative nutritional status of all patients after esophagectomy [17].

### Perioperative nutritional support and rehabilitation

According to our protocol, patients were instructed to abstain from smoking and alcohol consumption for at least 4 weeks before surgery. Physiotherapists commenced respiratory rehabilitation using an incentive spirometer. Following the surgery, early ambulation and restart of respiratory rehabilitation were encouraged. An elemental diet was started at 10 kcal/h from the day of surgery via a jejunostomy tube for patients with a jejunostomy. The tube-feeding dose was gradually increased to 1200 kcal/day. Enteral nutrition through jejunostomy was continued in the patients, even after the hospital discharge until there were satisfactory results in oral intake [17].

At the time of oral feeding initiation, the rehabilitation physician and the speech–language–hearing therapists performed videofluoroscopic (VF) and videoendoscopic (VE) examinations, especially for the patients receiving multidisciplinary team support. The meals were started with a dysphagia diet. The diet was changed to liquids in accordance with the improvement in the swallowing function. Furthermore, the total calorie intakes were based on decisions made by the multidisciplinary team conference. The calorie intake at the time of discharge was approximately 1500 kcal/day after introduction of multidisciplinary team support [17]. After the first visit in the outpatient clinic, the dietician evaluated the patients’ dietary intake and continued to give nutritional advice regularly.

### Follow-up

Postoperative follow-up was performed using CT every 6 months and EGD every year for 5 years after surgery. Recurrence-free survival (RFS) was calculated from the operation to the day of recurrence of esophageal cancer. Overall survival (OS) was calculated from the operation to the day of death due to esophageal cancer. Patients were followed up until death or until the end of the study (May 31, 2021). Patients who died of an illness unrelated to esophageal cancer, interrupted follow-up, or under following up were recognized as censored, and RFS and OS were calculated based on the days until censoring.

### Statistical analysis

The time-dependent ROC analysis was performed using R software v 4.0.5 (R Foundation for Statistical Computing, Vienna, Austria [19]). The time-dependent ROC analysis had used to diagnosis 5-year survival in this study. Other statistical analyses were performed using IBM SPSS Statistics version 26 for Windows (Chicago, IL, USA). Medians and ranges were calculated, and differences were identified using Student’s *t* test. The Mann–Whitney *U* test was used for non-parametric analyses. Differences between each category were identified using the Chi-square test or Fisher’s exact test, and the Kruskal–Wallis test was used to identify the differences in continuous variables. Survival curves were produced using the Kaplan–Meier survival method and log-rank test. Hazard ratios (HRs) were calculated, and univariate and multivariate analyses were performed using Cox proportional hazards regression models. The threshold for significance was set up at *p* < 0.05.

## Results

### The impact of preoperative PNI level on survival

Clinicopathological features between Group H and Group L are shown in Table 1. The median follow-up period was 43.4 (4.3–133.5) months. Patients in Group L had a more advanced clinical stage (*p* < 0.001) and, thus, the rate of patients who received neoadjuvant chemotherapy was higher in Group L (*p* = 0.004). The rate of multidisciplinary team support was similar in both groups (*p* = 0.736) (Table 1). Radical esophagectomy with two- or three-field LN dissection was performed in all patients. There was no significant difference in the rate of minimally invasive esophagectomy (*p* = 0.338). Moreover, no significant difference was observed in terms of LN dissection, operation time, and jejunostomy (Table 1). The rate of reconstruction organ was similar among both groups. With postoperative complications, the prevalence of postoperative pneumonia was significantly higher in Group L than that in Group H (39.1% vs. 23.6%, *p* = 0.038). There was no significant difference in AL and SSI between Groups L and H (AL 20.3% vs. 21.3%, *p* = 1.000; SSI 29.0% vs. 30.3%, *p* = 1.000). Pathological study showed that the patients in Group L were diagnosed at an advanced stage (*p* = 0.002). The rate of patients who received either adjuvant chemotherapy or radiation was higher in Group L than those in Group H (*p* = 0.012). There was no significant difference in the proportion of local recurrence between the groups. However, the rates of patients who had regional LN and distant organ recurrence were significantly higher in Group L than in Group H (regional LN recurrence: 31.9% vs 17.0%, *p* = 0.037; distant organ recurrence: 34.8% vs 17.0%, *p* = 0.015, respectively) (Table 1).

**Table 1:** Clinicopathological features between Groups H and L

	All cases, *n* = 158	Group H, *n* = 89	Group L, *n* = 69	*p* value
Age (median, years)†	67 (40–82)	67 (42–81)	67 (40–82)	0.702
Gender (%)				0.207
Male	140 (88.6%)	76 (85.4%)	64 (92.8%)	
Female	18 (11.4%)	13 (14.6%)	5 (7.2%)	
Preoperative body weight (median, kg)†	57.6 (36.0–84.4)	57.4 (36.0–80.3)	57.7 (36.6–84.4)	0.673
Preoperative BMI (median, kg/m^2^)†	21.1 (14.2–29.0)	21.3 (14.2–29.0)	20.7 (14.2–28.9)	0.142
Location of tumor (%)				0.943
Ut	16 (10.1%)	9 (10.1%)	7 (10.1%)	
Mt	89 (56.3%)	49 (55.1%)	40 (58.0%)	
Lt and Ae	53 (33.5%)	31 (34.8%)	22 (31.9%)	
Clinical stage, TNM 8th (%)				< 0.001
Stage I	65 (41.1%)	50 (56.2%)	15 (21.7%)	
Stage II	49 (31.0%)	24 (27.0%)	25 (36.2%)	
Stage III	39 (24.7%)	15 (16.9%)	24 (34.8%)	
Stage IVA	5 (3.2%)	0 (0.0%)	5 (7.2%)	
Preoperative therapy (%)				0.004
None	78 (49.4%)	53 (59.6%)	44 (63.8%)	
NAC	80 (50.6%)	36 (40.4%)	25 (36.2%)	
Multidisciplinary team support (%)	53 (33.5%)	31 (34.8%)	22 (31.9%)	0.736
Preoperative serum albumin†	4.1 (2.9–4.9)	4.2 (3.8–4.9)	3.7 (2.9–4.3)	< 0.001
Preoperative serum total lymphocyte count†	1425 (539–3501)	1575 (818–3501)	1271 (539–2366)	< 0.001
Preoperative PNI†	47.7 (32.2–62.7)	50.9 (46.9–62.7)	40.2 (20.9–51.3)	< 0.001
Surgical approach (%)				0.338
Thoracotomy	76 (48.1%)	46 (51.7%)	30 (43.5%)	
MIE	82 (51.9%)	43 (48.3%)	39 (56.5%)	
LN dissection (%)				0.802
2-field	18 (11.4%)	11 (12.4%)	7 (10.1%)	
3-field	140 (88.6%)	78 (87.6%)	62 (89.9%)	
Reconstruct organ (%)				0.105
Gastric conduit	148 (93.7%)	86 (96.6%)	62 (89.9%)	
Colon conduit	10 (6.3%)	3 (3.4%)	7 (10.1%)	
Jejunostomy (%)	78 (49.4%)	42 (47.2%)	36 (52.2%)	0.631
Operation time (median, min) †	603 (318–1008)	603 (347–1008)	618 (318–982)	0.611
Complications, C–D grade, ≥ 2 (%)				
All infectious complications	80 (50.6%)	42 (47.2%)	38 (55.1%)	0.341
AL	33 (20.9%)	19 (21.3%)	14 (20.3%)	1.000
Pneumonia	48 (30.4%)	21 (23.6%)	27 (39.1%)	0.038
SSI	47 (29.7%)	27 (30.3%)	20 (29.0%)	1.000
Pathological stage, TNM 8th (%)				0.002
Stage 0	4 (2.5%)	1 (1.1%)	3 (4.3%)	
Stage IA/IB	45 (28.5%)	36 (40.4%)	9 (13.0%)	
Stage IIA/IIB	34 (21.5%)	18 (20.2%)	16 (23.2%)	
Stage IIIA/IIIB	50 (31.7%)	23 (25.8%)	27 (39.1%)	
Stage IVA/IVB	25 (15.8%)	11 (12.4%)	14 (20.3%)	
Adjuvant therapy (%)				0.012
None	92 (58.2%)	59 (66.3%)	32 (46.4%)	
Chemotherapy	65 (63.3%)	29 (32.6%)	37 (53.6%)	
Radiation	1 (0.6%)	1 (1.1%)	0 (0.0%)	
POM1 serum albumin†	3.5 (1.6–4.6)	3.6 (2.0–4.6)	3.5 (1.6–4.4)	0.014
POM1 serum total lymphocyte count†	1183 (300–3107)	1261 (402–3107)	1023 (300–2357)	0.003
POM1 PNI†	41.1 (20.9–56.5)	42.9 (23.4–56.5)	40.2 (20.9–51.3)	0.003
Recurrence site* (%)				
Local	12 (7.6%)	5 (5.7%)	7 (10.1%)	0.369
Regional LN	37 (23.6%)	15 (17.0%)	22 (31.9%)	0.037
Distant organ	39 (24.8%)	15 (17.0%)	24 (34.8%)	0.015
Death unrelated to esophageal cancer (%)	8 (5.1%)	6 (6.7%)	2 (2.9%)	0.467

*BMI* body mass index, *Ut* upper thoracic esophagus, *Mt* middle thoracic esophagus, *Lt* lower thoracic esophagus, *Ae* abdominal esophagus, *NAC* neoadjuvant chemotherapy, *PNI* prognostic nutritional index, *MIE* minimal invasive esophagectomy, *LN* lymph node, *C–D* Clavien–Dindo, *AL* anastomotic leakage, *SSI* surgical site infection, *POM* postoperative month

^†^Values are presented as median (range)

^*^Some patients were existed multiple sites of recurrence

**Fig. 2:**
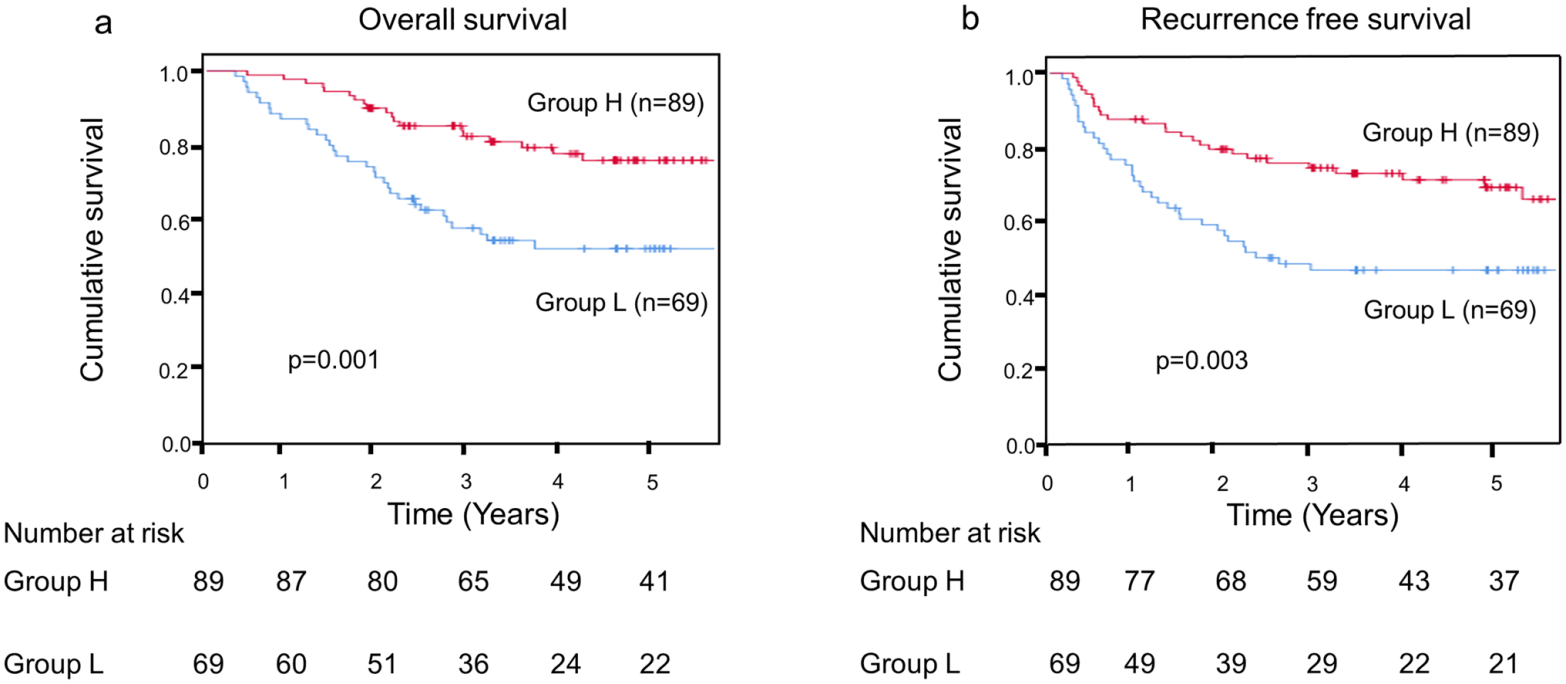
Kaplan–Meier analysis with preoperative prognostic nutritional index levels. **a** Comparison of overall survival. **b** Comparison of recurrence free survival

In Kaplan–Meier analysis, the OS and RFS were significantly worse in Group L than in Group H (*p* = 0.001, *p* = 0.003) (Fig. 2).

### The impact of changes in PNI level from preoperative to postoperative phase on survival

To confirm the correlation between the changes in PNI level from the preoperative to postoperative phase and survival, the survival rates among the three groups were compared. Clinicopathological features between Groups L–H and L–L are shown in Table 2. There was no significant difference in clinical stage between Groups L–H and L–L (*p* = 0.132). The proportion of multidisciplinary team support in Group L-L tended to be lower than that in Group L–H (25.6% vs. 40.0%, *p* = 0.298). The incidence of postoperative infectious complications (AL, pneumonia, and SSI) was higher in Group L–L than in Group L–H (*p* = 0.032). The prevalence of AL and SSI was significantly higher in Group L–L than those in Group L–H (AL: 30.8% vs. 6.7%, *p* = 0.016; SSI: 41.0% vs. 13.3%, *p* = 0.016). The rate of patients who received enteral nutrition in the early postoperative phase was similar between the groups (53.3% in Group L–H vs. 51.3% in Group L–L, *p* = 1.000). There was no difference in the population of patients with death unrelated to esophageal cancer (*p* = 1.000). The proportion of patients who received adjuvant chemotherapy was similar between the two groups (*p* = 0.336). However, adjuvant therapy was suspended in two patients from Group L–L due to adverse events (Table 2).

**Table 2:** Clinicopathological features between Groups L–H and L–L

	Group L–H, *n* = 30	Group L–L, *n* = 39	*p* value
Age (median, years)†	66.5 (48–76)	67 (40–82)	0.216
Gender (%)			0.159
Male	26 (86.7%)	38 (97.4%)	
Female	4 (13.3%)	1 (2.6%)	
Location of tumor (%)			0.593
Ut	2 (6.7%)	5 (12.8%)	
Mt	17 (56.7%)	23 (59.0%)	
Lt and Ae	11 (36.7%)	11 (28.2%)	
Clinical stage, TNM 8th (%)			0.132
Stage I	10 (33.3%)	5 (12.8%)	
Stage II	8 (26.7%)	17 (43.6%)	
Stage III	11 (36.7%)	13 (33.3%)	
Stage IVA	1 (3.3%)	4 (10.3%)	
Preoperative therapy (%)			1.000
None	11 (36.7%)	14 (35.9%)	
NAC	19 (63.3%)	25 (64.1%)	
Multidisciplinary team support (%)	12 (40.0%)	10 (25.6%)	0.298
Surgical approach (%)			1.000
Thoracotomy	13 (43.3%)	17 (43.6%)	
MIE	17 (56.7%)	22 (56.4%)	
LN dissection (%)			0.128
2-field	1 (3.3%)	6 (15.4%)	
3-field	29 (96.7%)	33 (84.6%)	
Reconstruct organ (%)			0.690
Gastric conduit	26 (86.7%)	36 (92.3%)	
Colon conduit	4 (13.3%)	3 (7.7%)	
Jejunostomy (%)	16 (53.3%)	20 (51.3%)	1.000
Operation time (median, min) †	559.5 (318–982)	645 (441–927)	0.055
Complications, C–D grade, ≥ 2 (%)			
All infectious complications	12 (40.0%)	26 (66.7%)	0.032
AL	2 (6.7%)	12 (30.8%)	0.016
Pneumonia	10 (33.3%)	17 (43.6%)	0.460
SSI	4 (13.3%)	16 (41.0%)	0.016
Pathological stage, TNM 8th (%)			0.320
Stage 0	3 (10.0%)	0 (0.0%)	
Stage IA/IB	5 (16.7%)	4 (10.3%)	
Stage IIA/IIB	6 (20.0%)	10 (25.6%)	
Stage IIIA/IIIB	11 (36.7%)	16 (37.9%)	
Stage IVA/IVB	5 (16.7%)	9 (23.1%)	
Adjuvant therapy (%)			0.336
None	13 (43.3%)	22 (56.4%)	
Chemotherapy	17 (56.7%)	17 (43.6%)	
POM1 serum albumin†	3.8 (3.3–4.4)	3.0 (1.6–3.7)	< 0.001
POM1 serum total lymphocyte count†	1218.5 (590–2357)	972 (300–2244)	0.011
POM1 PNI†	44.5 (40.9–51.3)	35.1 (20.9–40.8)	< 0.001
Recurrence site* (%)			
Local	2 (6.7%)	5 (12.8%)	0.690
Regional LN	6 (20.0%)	16 (41.0%)	0.074
Distant organ	8 (26.7%)	16 (41.0%)	0.308
Death unrelated to esophageal cancer (%)	1 (3.3%)	1 (2.6%)	1.000

*Ut* Upper thoracic esophagus, *Mt* middle thoracic esophagus, *Lt* lower thoracic esophagus; *Ae* Abdominal esophagus, *NAC* neoadjuvant chemotherapy, *MIE* minimal invasive esophagectomy, *LN* lymph node, *C–D* Clavien–Dindo, *AL* anastomotic leakage, *SSI* surgical site infection, *POM* postoperative month, *PNI* prognostic nutritional index

^†^Values are presented as median (range)

^*^Some patients were existed multiple sites of recurrence

**Fig. 3:**
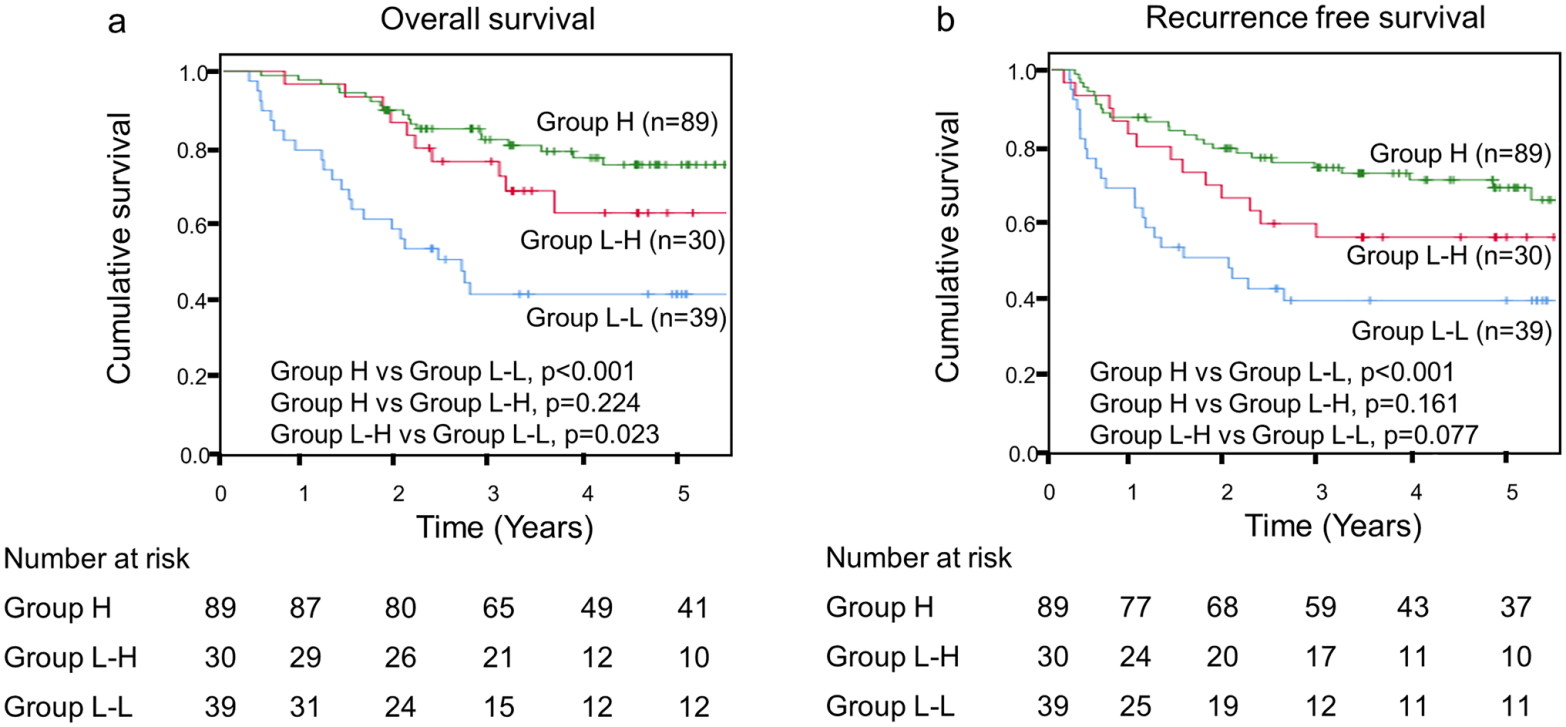
Kaplan–Meier analysis of the changes in prognostic nutritional index levels from preoperative to postoperative phase. **a** Comparison of overall survival between the three groups. **b** Comparison of recurrence free survival between the three groups

In the Kaplan–Meier analysis, the RFS in Group L–H tended to be better than those in Group L–L (*p* = 0.077). Furthermore, patients in Group L–H had significantly better OS than those in Group L–L (*p* = 0.023) (Fig. 3). Patients in Group L–L had significantly worse OS and RFS than those in Group H (*p* < 0.001, *p* < 0.001). However, there was no significant difference in OS and RFS between Groups L–H and H (*p* = 0.224, *p* = 0.161) (Fig. 3).

### Risk factors for poor prognosis after esophagectomy

All of the variables shown in Table 3 were potentially significant clinicopathologic variables of prognosis in patients with ESCC. In univariate analysis, neoadjuvant chemotherapy, advanced pathological stage (≥ II), and Group L–L were associated with a worse OS (*p* = 0.004, *p* < 0.001, and *p* < 0.001, respectively) (Table 3). Postoperative complications (AL, pneumonia, and SSI) were not associated with OS (*p* = 0.548) (Table 3). In multivariate analysis, advanced pathological stage (HR 10.947; 95% CI 2.590–46.268; *p* = 0.001) and Group L-L (HR 2.171; 95% CI 1.249–3.775; *p* = 0.006) were independent predictors of worse OS (Table 3).

**Table 3:** Independent factors of clinicopathological, surgical, and pathological features on shorter overall survival

	Univariate analysis			Multivariate analysis		
	HR	*p*	95% CI	HR	*p*	95% CI
Age (> 66 vs ≤ 66)	0.962	0.889	0.561–1.650			
Transthoracic approach (thoracotomy vs MIE)	1.041	0.883	0.607–1.785			
Reconstruction (colon conduit vs gastric conduit)	2.093	0.090	0.891–4.915			
All infectious complications (+ vs −)	1.181	0.548	0.686–2.035			
AL (+ vs −)	1.114	0.736	0.595–2.084			
Pneumonia (+ vs −)	1.518	0.138	0.874–2.635			
SSI (+ vs −)	1.224	0.481	0.698–2.147			
Adjuvant therapy (+ vs −)	1.383	0.238	0.807–2.371			
Neoadjuvant chemotherapy (+ vs −)	2.301	0.004	1.302–4.067	1.353	0.306	0.758–2.416
Pathological stage (≥ II vs < II)	14.803	< 0.001	3.601–60.857	10.947	0.001	2.590–46.268
Change of PNI level (Group L–L vs other groups)	3.022	< 0.001	1.746–5.228	2.171	0.006	1.249–3.775

*HR* hazard ratio, *CI* confidence interval, *MIE* minimally invasive esophagectomy, *AL* anastomotic leakage, *SSI* surgical site infection, *PNI* prognostic nutritional index

### Effect of multidisciplinary team support

A stratified analysis before and after introduction of multidisciplinary team support was performed. The proportion of patients in Group L–H increased markedly from 17.1% before initiation of the multidisciplinary team support to 22.6% after initiation of the multidisciplinary team support (Online Resource 3). Before introduction of multidisciplinary team support, patients in Group L–H showed significantly better OS and RFS than those in Group L–L (median survival time (MST); OS: 61.1 months vs. 35.4 months; RFS: 61.1 months vs. 24.4 months). Furthermore, there was no difference in the OS and RFS between Groups L–H and H. Although the number of patients who received multidisciplinary team support was small, after it was initiated, patients in Group L–H also tended to have a good OS than those in Group L–L (MST of OS: 36.2 months vs. 23.7 months). Moreover, the survival curves were also similar between Groups L–H and H (Online Resource 3).

### Survival impact of postoperative nutritional support for patients with infectious complications

To investigate the survival impact postoperative nutritional support, the long-term outcomes of patients with postoperative infectious complications (AL, pneumonia, and SSI) were assessed using a stratified analysis which patients were divided into groups based on whether they developed postoperative infectious complications (Online Resource 4). In the stratified analysis, either with or without postoperative infectious complications, patients in Group L–H were tended to have better OS and RFS than those in Group L–L (Online Resource 4).

## Discussion

This study demonstrated that preoperative-low PNI patients who did not sufficiently recover in terms of postoperative PNI level had a significantly worse OS. In contrast, preoperative-low PNI patients who sufficiently recovered in terms of postoperative PNI levels had similar OS to those with preoperative-high PNI levels. These results suggested that the changes in PNI levels from the pre- to postoperative phase may predict the prognosis in patients with ESCC, and successful recovery of PNI level after transthoracic esophagectomy can improve the OS in preoperative-low PNI patients to match that of preoperative-high PNI patients.

PNI was initially proposed by Buzby [20] and modified by Onodera [12]. PNI, which includes albumin level and lymphocyte counts in its calculation, is a combined simple index of nutrition and immune status. Low albumin levels and lymphocyte counts reflect systemic inflammation in cancer patients. For several types of cancer, including pancreatic cancer, hepatocellular carcinoma, gastric cancer, and colon cancer, PNI was shown to be a significant prognostic factor. Previous studies have reported that preoperative PNI level was associated with prognosis for ESCC [14, 21, 22]. However, to the best of our knowledge, this is the first report on the survival impact of the changes in PNI level during the perioperative period after esophagectomy. In this study, postoperative PNI was set from blood test of 1 month later. At acute phase after surgery, intensive invasion and systemic inflammation persisted, and these factors might inhibit the effect of nutritional support. In contrast, some patients in this study received adjuvant therapy within 3 months after surgery, which might affect the PNI level.

The controlling nutritional status (CONUT) score is another representative nutritional index which reported as an independent predictor of prognosis of esophageal cancer [23]. Evaluation of the association between the preoperative PNI levels and the preoperative CONUT score was performed in this dataset. In this study, the preoperative PNI levels was significantly lower in the high CONUT score group (CONUT score 2 or higher) than those in the low CONUT score group (CONUT score 0–1) (data not shown). These results suggested that there was a correlation between both nutritional indexes. On the other hand, Lin, et al. compared the association of the PNI level and CONUT score with prognosis of gastric cancer patients, and reported that the CONUT score was important in assessing the risk of severe postoperative morbidity, and the PNI level associated more precisely with long-term prognosis [24]. That is why the PNI level was used as an indicator in this study.

In this study, patients in Group L–L tended to have more frequent postoperative complications than those in Group L–H. Postoperative complications reportedly induced the suppression of albumin production and elevation of white blood cells, particularly neutrophils, resulting in insufficient PNI recovery [25]. Interestingly, the stratified analysis for patients with postoperative infectious complications demonstrated that patients in Group L–L were tended to have poor OS and RFS than those in Group L–H. Furthermore, there was no correlation between postoperative complications and OS from the univariable Cox regression analysis. These results suggested that preventing a decline of PNI level could improve the prognosis, even in patients with postoperative complications.

The proportion of patients in Group L–H increased after introduction of multidisciplinary team support. In the stratified analysis, patients in Group L–L were tended to have poor OS than those in Group L–H both before and after introduction of multidisciplinary team support. These results indicated that intensive postoperative care with a multidisciplinary team support could contribute not only to the prevention of postoperative complications and maintenance of PNI levels, but also to the prolongation of prognosis, especially in preoperative-low PNI level patients. Moreover, two patients in Group L-L stopped adjuvant chemotherapy. These cases suggested that sufficient PNI recovery after esophagectomy improved tolerance to adjuvant chemotherapy. In multidisciplinary team support, team conference in which the members could share the patients’ nutritional status, swallowing function from VF and VE, and calorie intake could have contributed to the enhanced nutritional support [17]. Moreover, regular nutritional counseling in outpatient clinic also could support patients’ nutritional status [17].

Patients in Group L–L had significantly worse RFS than the others did, and patients in Group L–H had similar RFS to those in Group H. However, when limited to the first year after esophagectomy, the decline in RFS rate was similar between Groups L–H and L–L. After a year, the RFS rate continued to decline in Group L–L, but that in Group L–H was relatively stable. These results suggested that postoperative PNI reflected tumor immunity. Okadome et al. reported that the systemic nutritional and immunological status in patients may be associated with local tumor immunity by evaluating the relationship between PNI and tumor-infiltrating lymphocyte status [13].

The study had some limitations. First, this was a retrospective study conducted in a single institution. However, this study reviewed consecutive patients at our institution, which reduced selection bias. Another limitation is that the study period was relatively long. Consequently, the treatment of patients with ESCC, including the maturation of surgical techniques, regimen of neoadjuvant chemotherapy, and perioperative management, could have changed over this period. There were some biases in the operation and perioperative interventions. Hence, a multi-institutional prospective study must be conducted to validate the current results.

In conclusion, the recovery of PNI levels after esophagectomy could improve the prognosis in patients with preoperative malnutrition. Further studies are warranted to validate this classification and investigate the survival benefit of PNI changes during esophagectomy.

## Supplementary Information

Below is the link to the electronic supplementary material.

Supplementary file1 (PDF 132 kb)

Supplementary file2 (PDF 126 kb)

Supplementary file3 (PDF 264 kb)

Supplementary file4 (PDF 235 kb)
