# Supplementary material for: Survival impact of perioperative changes in prognostic nutritional index levels after esophagectomy
Source: Esophagus. 2021 Sep 21;19(2):250–9. doi: 10.1007/s10388-021-00883-5 (PMC8921021; doi:10.1007/s10388-021-00883-5)
Supplement: Supplementary file 1 — Supplementary file1 (PDF 132 kb) [file 10388_2021_883_MOESM1_ESM.pdf]

Online Resource 1

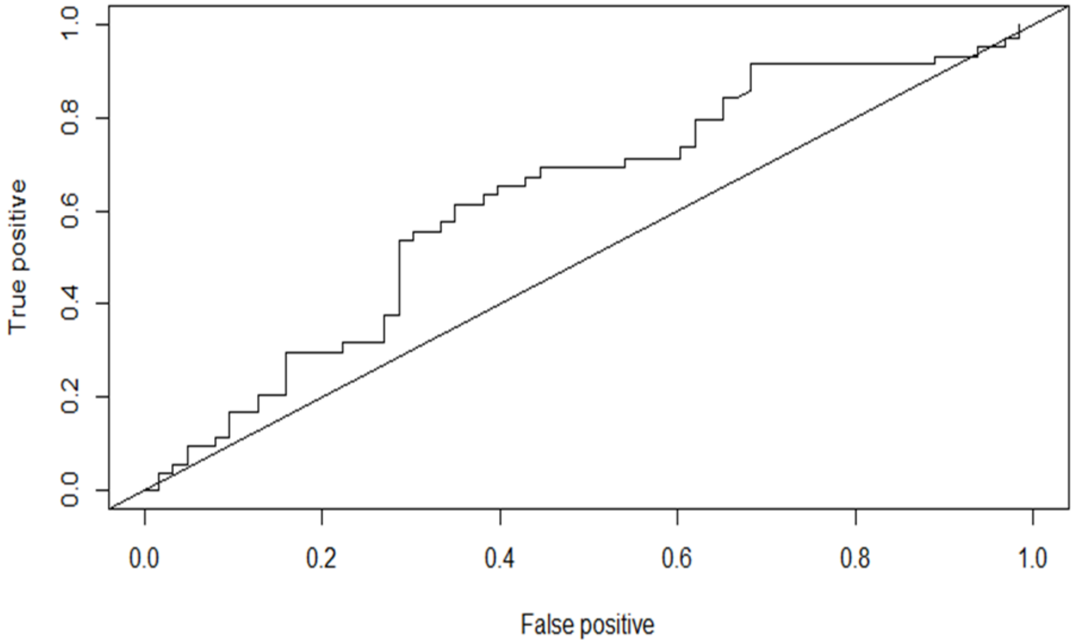

Online Resourse 1 The time-dependent ROC analysis of preoperative PNI predicting mortality. The cutoff value was 46.9 (sensitivity = 0.613, specificity = 0.651).

Title: Survival impact of perioperative changes in prognostic nutritional index levels after esophagectomy

Journal name: Esophagus

Ryoma Haneda, MD[1], Yoshihiro Hiramatsu, MD, PhD[1,2], Sanshiro Kawata, MD, PhD[1], Junko Honke, RN, MSN[2], Wataru Soneda, MD[1], Tomohiro Matsumoto, MD[1], Yoshifumi Morita, MD, PhD[1], Hirotoshi Kikuchi, MD, PhD[1], Kinji Kamiya, MD, PhD[1], Hiroya Takeuchi, MD, PhD[1]

[1]Department of Surgery, Hamamatsu University School of Medicine, Hamamatsu, Japan

[2]Department of Perioperative Functioning Care and Support, Hamamatsu University School of Medicine, Hamamatsu, Japan

Corresponding author: Yoshihiro Hiramatsu, MD, PhD.

Department of Perioperative Functioning Care and Support, Hamamatsu University School of Medicine, 1-20-1 Handayama, Higashi-ku, Hamamatsu, Shizuoka 431-3192, Japan

E-mail: hiramatu@hama-med.ac.jp

Phone: +81-53-435-2427; Fax: +81-53-435-2428
