# Supplementary material for: Survival impact of perioperative changes in prognostic nutritional index levels after esophagectomy
Source: Esophagus. 2021 Sep 21;19(2):250–9. doi: 10.1007/s10388-021-00883-5 (PMC8921021; doi:10.1007/s10388-021-00883-5)
Supplement: Supplementary file 3 — Supplementary file3 (PDF 264 kb) [file 10388_2021_883_MOESM3_ESM.pdf]

Online Resource 3

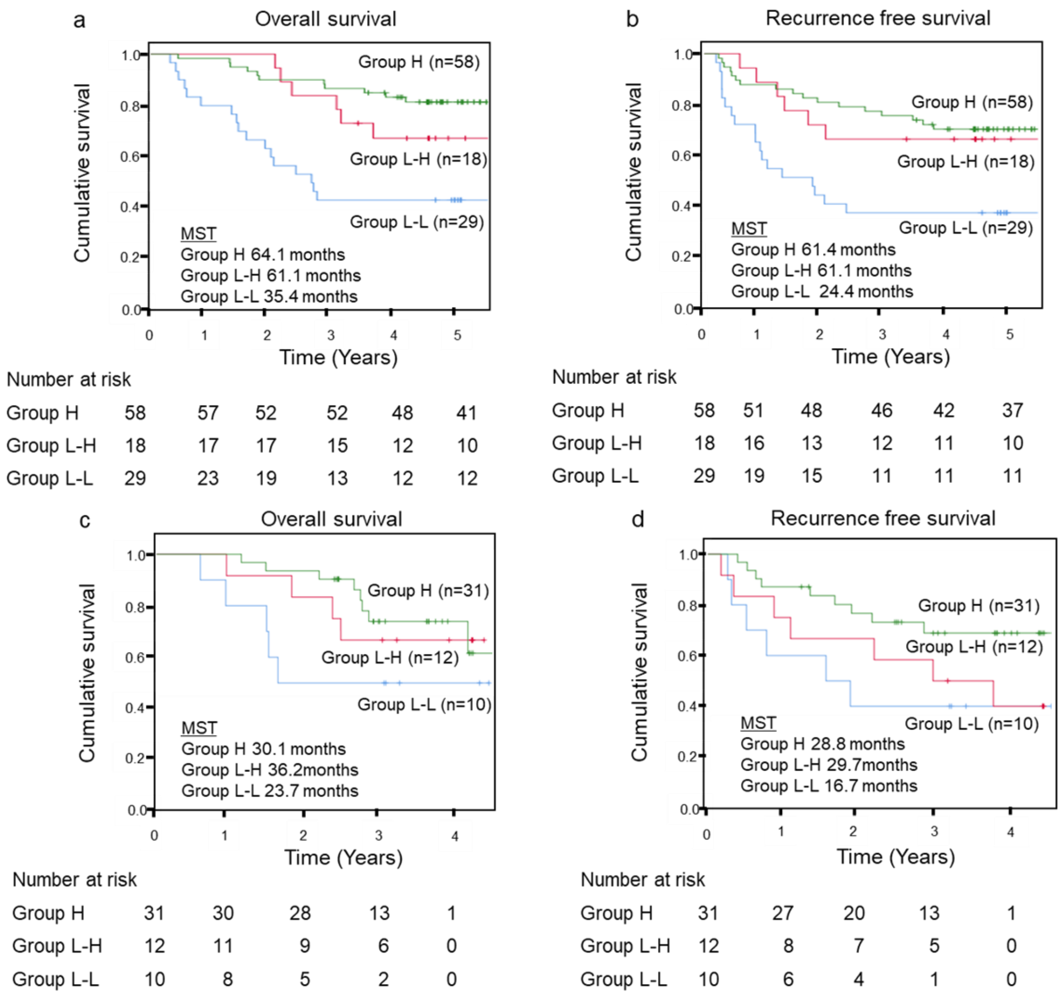

a) Number at risk

Group H	58	57	52	52	48	41
Group L-H	18	17	17	15	12	10
Group L-L	29	23	19	13	12	12

b) Number at risk

Group H	58	51	48	46	42	37
Group L-H	18	16	13	12	11	10
Group L-L	29	19	15	11	11	11

c) Number at risk

Group H	31	30	28	13	1
Group L-H	12	11	9	6	0
Group L-L	10	8	5	2	0

d) Number at risk

Group H	31	27	20	13	1
Group L-H	12	8	7	5	0
Group L-L	10	6	4	1	0

Online Resource 3 Stratified analysis before and after multidisciplinary team support. (a) Comparison of overall survival between the three groups before multidisciplinary team support (b) Comparison of recurrence free survival between the three groups before multidisciplinary team support (c) Comparison of overall survival between the three groups after multidisciplinary team support (d) Comparison of recurrence free survival between the three groups after multidisciplinary team support. MST, Median survival time

Title: Survival impact of perioperative changes in prognostic nutritional index levels after esophagectomy

Journal name: Esophagus

Ryoma Haneda, MD[1], Yoshihiro Hiramatsu, MD, PhD[1,2], Sanshiro Kawata, MD, PhD[1], Junko Honke, RN, MSN[2], Wataru Soneda, MD[1], Tomohiro Matsumoto, MD[1], Yoshifumi Morita, MD, PhD[1], Hirotoshi Kikuchi, MD, PhD[1], Kinji Kamiya, MD, PhD[1], Hiroya Takeuchi, MD, PhD[1]

[1]Department of Surgery, Hamamatsu University School of Medicine, Hamamatsu, Japan

[2]Department of Perioperative Functioning Care and Support, Hamamatsu University School of Medicine, Hamamatsu, Japan

Corresponding author: Yoshihiro Hiramatsu, MD, PhD.

Department of Perioperative Functioning Care and Support, Hamamatsu University School of Medicine, 1-20-1 Handayama, Higashi-ku, Hamamatsu, Shizuoka 431-3192, Japan

E-mail: hiramatu@hama-med.ac.jp

Phone: +81-53-435-2427; Fax: +81-53-435-2428
